# GGC Repeat Expansion in the *NOTCH2NLC* Gene Is Associated With a Phenotype of Predominant Motor–Sensory and Autonomic Neuropathy

**DOI:** 10.3389/fgene.2021.694790

**Published:** 2021-07-07

**Authors:** Hui Wang, Jiaxi Yu, Meng Yu, Jianwen Deng, Wei Zhang, He Lv, Jing Liu, Xin Shi, Wei Liang, Zhirong Jia, Daojun Hong, Lingchao Meng, Zhaoxia Wang, Yun Yuan

**Affiliations:** ^1^Department of Neurology, Peking University First Hospital, Beijing, China; ^2^Beijing Key Laboratory of Neurovascular Disease Discovery, Beijing, China; ^3^Department of Neurology, The First Affiliated Hospital of Nanchang University, Nanchang, China

**Keywords:** inherited peripheral neuropathy, motor–sensory and autonomic neuropathy, *NOTCH2NLC*, GGC repeat expansions, *NOTCH2NLC*-related repeat expansion spectrum

## Abstract

There is still a considerable proportion of patients with inherited peripheral neuropathy (IPN) whose pathogenic genes are unknown. This study was intended to investigate whether the GGC repeat expansion in the *NOTCH2NLC* is presented in some patients with IPN. A total of 142 unrelated mainland Chinese patients with highly suspected diagnosis of IPN without any known causative gene were recruited. Repeat-primed polymerase chain reaction (RP-PCR) was performed to screen GGC repeat expansion in *NOTCH2NLC*, followed by fluorescence amplicon length analysis-PCR (AL-PCR) to determine the GGC repeat size. Detailed clinical data as well as nerve, muscle, and skin biopsy were reviewed and analyzed in the *NOTCH2NLC*-related IPN patients. In total, five of the 142 patients (3.52%) were found to have pathogenic GGC expansion in *NOTCH2NLC*, with repeat size ranging from 126 to 206 repeats. All the *NOTCH2NLC*-related IPN patients presented with adult-onset motor–sensory and autonomic neuropathy that predominantly affected the motor component of peripheral nerves. While tremor and irritating dry cough were noted in four-fifths of the patients, no other signs of the central nervous system were presented. Electrophysiological studies revealed both demyelinating and axonal changes of polyneuropathy that were more severe in lower limbs and asymmetrically in upper limbs. Sural nerve pathology was characterized by multiple fibers with thin myelination, indicating a predominant demyelinating process. Muscle pathology was consistent with neuropathic changes. P62-positive intranuclear inclusions were observed in nerve, skin, and muscle tissues. Our study has demonstrated that GGC expansion in *NOTCH2NLC* is associated with IPN presenting as predominant motor–sensory and autonomic neuropathy, which expands the phenotype of the *NOTCH2NLC*-related repeat expansion spectrum. Screening of GGC repeat expansions in the *NOTCH2NLC* should be considered in patients presenting with peripheral neuropathy with tremor and irritating dry cough.

## Introduction

Inherited peripheral neuropathy (IPN) represents a large heterogenous group of hereditary diseases including hereditary motor and sensory neuropathy (HMSN), more commonly called Charcot–Marie–Tooth disease (CMT), hereditary sensory and autonomic neuropathy (HSAN), distal hereditary motor neuropathy (dHMN), and small fiber neuropathies (SFN) (Schröder and Matthiesen, 1985; Eggermann et al., 2018). There are also other forms of IPN such as transthyretin familial amyloid polyneuropathy (TTR-FAP), a severe hereditary neuropathy affecting the sensorimotor and autonomic function as well as other organs caused by mutation of the TTR gene (Adams et al., 2017; Du et al., 2021). With the development of next-generation sequencing (NGS) technologies, including disease-target gene panels, whole-exome sequencing (WES), whole-genome sequencing (WGS), and high-throughput transcriptome sequencing, more than 100 and 30 genes have been associated with CMT and dHMN, respectively (Laurá et al., 2019; Pipis et al., 2019; Liu et al., 2020). However, there are still a large proportion of patients who are left unexplained from a genetic perspective.

Recently, GGC repeat expansion in the *NOTCH2NLC* has been identified as the pathogenic cause of neuronal intranuclear inclusion disease (NIID), a progressive neurodegenerative disorder, which is pathologically characterized by the presence of eosinophilic hyaline intranuclear inclusion in the central and peripheral nerves system, as well as in other tissues (Deng et al., 2019; Ishiura et al., 2019; Sone et al., 2019; Tian et al., 2019). Based on the age of onset, NIID can be divided into infantile, juvenile, and adult clinical subgroups (Takahashi-Fujigasaki, 2003). Adult-onset NIID patients have a wide range of clinical manifestations including dementia, encephalitic episode, Parkinsonism, cerebellar ataxia, autonomic dysfunction, stroke-like episodes, and muscle weakness (Sone et al., 2016; Deng et al., 2019; Tian et al., 2019). Despite the findings of previous studies that some NIID patients showed peripheral neuropathy, the role of genetics has not been elucidated (Sone et al., 2005). Given the clinical and pathological overlapping of NIID and IPN, it can be inferred that some of the patients with GGC expansion in the *NOTCH2NLC* may manifest symptoms predominantly limited to peripheral neuropathy and in the form of hereditary motor–sensory and autonomic neuropathy. Therefore, we aimed to detect the GGC repeat expansion in *NOTCH2NLC* in a cohort of patients with genetically undetermined IPN.

## Materials and Methods

### Patients and Materials

A cohort of 142 unrelated patients from mainland China were recruited in this study, who were referred to the Department of Neurology, Peking University First Hospital, between 2011 and 2020. The inclusion criteria for patients included: (1) clinical diagnosis with or highly suspected IPN, characterized by motor, sensory, or motor–sensory form, with a slow disease progression of more than 6 months; (2) exclusion of acquired causes of neuropathy such as endocrine, immunologic, infectious, metabolic, nutritional, drug and substance-related, connective tissue, and paraneoplastic; and (3) exclusion of known causative genes of CMT, dHMN, and TTR-FAP by MLPA, targeted NGS, and Sanger sequencing. Among these 142 patients, the ratio of males to females was 103:39. Twenty-three of the patients had positive family history, and 119 were sporadic. Control subjects (*n* = 100) were collected from Peking University First Hospital.

### Polymerase Chain Reaction Analysis

We combined repeat-primed polymerase chain reaction (RP-PCR) and fluorescence amplicon length analysis-PCR (AL-PCR) to analyze the number of GGC repeats in *NOTCH2NLC*, as described in a previously reported method (Deng et al., 2019; Deng et al., 2021). First, RP-PCR was performed in all individuals to find out whether they carried any pathogenic expansion. The primer mix of RP-PCR contained three primers: NOTCH2NLC-F, 5′-FAM-GGCATTTGCGCCTGTGCTTCGGACCGT-3′; M13 -(GGC)4(GGA)2R, 5′-CAGGAAACAGCTATGACCTCCTCCG CCGCCGCCGCC-3′; and M13-linker-R, 5′-CAGGAAACAG CTATGACC-3′. Then, to determine the accurate sizes of expanded repeats, AL-PCR was conducted in samples that showed a typical sawtooth pattern on RP-PCR. For AL-PCR, different primer pairs were used: NOTCH2NLC-AL-F, 5′-FAM-GGCATTTGCGCCTGTGCTTCGGACCGT-3’; NOTCH2NLC-AL-R, 5′-AGAGCGGCGCAGGGCGGGCATCTT-3′. In the present study, we applied cutoff values for the pathogenic and normal ranges of GGC repeat expansion as were used in previous studies (Ishiura et al., 2019; Tian et al., 2019; Yuan et al., 2020; Shi et al., 2021): ≥ 60 repeats as pathogenic expansion, ≤ 40 as normal repeat numbers, and 41–59 as intermediate repeat numbers.

### Clinical Investigation

Clinical history, systematic clinical examinations, and evaluations were collected in all the patients who had abnormal GGC repeat expansion in *NOTCH2NLC* identified. Clinical neurological and neuropsychological tests were conducted, including laboratory finding, electrophysiological studies, nerve ultrasound, brain magnetic resonance imaging (MRI), diffusion-weighted imaging (DWI), the Mini-Mental State Examination (MMSE), and the Montreal Cognitive Assessment (MoCA).

### Histology, Immunohistochemistry, and Electron Microscopic Studies

We re-reviewed the pathology of peripheral tissue biopsies that were prepared during diagnosis. Peripheral nerve, muscle, and skin biopsies were conducted following routine procedures. Specifically, we collected samples of the sural nerves from patients 2, 3, 4, and 5, and gastrocnemius muscle and skin from patients 3 and 4. Nerve and skin specimens were fixed in 2–3% glutaraldehyde, postfixed in 1% osmium tetroxide, dehydrated through serial alcohol baths, and embedded in Epon 812. Sections of samples (6 μm) were stained with hematoxylin and eosin (H&E), and semithin sections were stained with toluidine blue for light microscopy (Li et al., 2019). Myelinated fiber morphometry was performed as was described in a previous study (Herrmann et al., 1999). The sections were immunohistochemically stained with anti-p62 antibody (Abcam, ab56416). Ultrathin sections were contrasted with uranyl acetate and lead citrate before being examined under electron microscopy (Deng et al., 2020). Muscle samples were frozen and fixed in liquid nitrogen. Sections of all samples (8 μm) were stained with H&E, modified Gomori trichrome (mGT), NADH-tetrazolium reductase (NADH-TR), and adenosine triphosphatase (ATPase), as previously reported (Zhao et al., 2020).

### Immunofluorescence Study

Immunofluorescence staining was conducted on nerve, skin, and muscle sections with an anti-p62 antibody (Abcam, ab56416). The nuclei were counter-stained with DAPI. The images were acquired with a confocal microscope (Nikon A1MP) at × 60 magnification.

### Statistical Analysis

Statistical analysis was performed using SPSS version 25.0 (IBM Corp, Armonk, NY, United States) and Prism 8.0 software (GraphPad Software). Quantitative variables are presented as the mean ± standard deviations or median (interquartile range) and compared using Student’s *t*-test or Mann-Whitney test. Two-sided tests were used, and *p* < 0.05 was considered significant.

## Results

### Expanded GGC Repeats in *NOTCH2NLC* Were Identified in Inherited Peripheral Neuropathy

**FIGURE 1:**
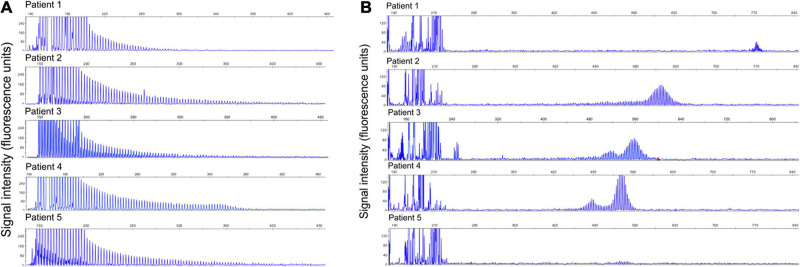
Validation of GGC repeat expansions and repeat lengths in the *NOTCH2NLC* gene. **(A)** Validation of GGC repeat expansions in the *NOTCH2NLC* gene among five index patients by RP-PCR. **(B)** Validation of repeat lengths of GGC repeats in the *NOTCH2NLC* gene among five index patients by AL-PCR.

Five patients had abnormal expansions that showed the typical sawtooth tail pattern on RP-PCR (Figure 1A). The analysis of expanded repeats size using AL-PCR revealed that all the patients had pathogenic expansions, with repeat sizes of 206, 151, 135, 129, and 126, respectively (Figure 1B). Hence, 3.52% (5/142) of the IPN patients in our cohort were found to have GGC expansions in the *NOTCH2NLC* gene.

Additionally, we conducted AL-PCR in 103 of these IPN patients without GGC repeats of *NOTCH2NLC* in the group and found that the median repeat number was 16 (7) (ranging from 6 to 35). The median repeat number of 100 normal controls was 16 (8) (ranging from 6 to 26). There was no significant difference between these two groups (*p* = 0.114). The distribution of repeat sizes in these 103 IPN patients and normal controls is described in Supplementary Figure 1.

### Clinical Features of *NOTCH2NLC*-Related Inherited Peripheral Neuropathy Patients

**FIGURE 2:**
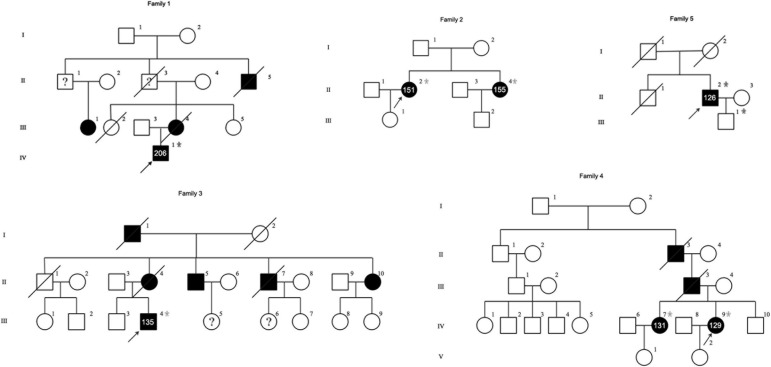
Pedigrees of five unrelated families with the neuropathy phenotype. The squares represent males and the circles represent females. Unaffected and affected individuals are marked by white and black symbols, respectively. The arrow indicates the index. A diagonal line through a symbol indicates a deceased individual. Asterisks indicate patients with available genomic DNA obtained from peripheral blood leukocytes and the number of GGC repeats is indicated. A question mark indicates the patient has suspicious clinical symptoms but without details.

The clinical features of the five IPN patients with abnormal GGC repeat expansions in *NOTCH2NLC* are listed in Table 1. Among them, four had family history, while one was sporadic (Figure 2).

**TABLE 1:** Clinical features of patients with abnormal GGC repeat expansions.

**Number**	**Patient 1**	**Patient 2**	**Patient 3**	**Patient 4**	**Patient 5**
ID	F1-IV:1	F2-II:2	F3-III:4	F4-IV:9	Sporadic
Gender	Male	Female	Male	Female	Male
Age (years)	42	45	39	50	65
Onset age (years)	28	29	27	32	59
Repeat size	206	151	135	129	126
Initial symptom	Muscle weakness in four limbs	Walking difficulty	Numbness in bilateral feet	Muscle weakness in distal four limbs	Numbness in left finger
Dysphagia	+	–	–	–	+
Muscle weakness	LD > LP > UD = UP	LD > LP	UD	LD > LP = UD = UP	UP > UD > LP = LD
Limb muscle atrophy	Diffuse	Distal	Distal	Diffuse	UD
Foot deformity	–	Pes cavus	–	–	–
Deep tendon reflex	↓↓	↓	↓↓	↓↓	↓↓
Pyramidal signs	–	–	–	–	–
Numbness	U + L	–	U + L	U (left hand)	U + L
Pain sensation	L	L (left foot)	U + L	–	U + L
Vibration sensation	–	–	–	–	–
Autonomic dysfunction					
-Bladder dysfunction	+	+	+	+	+
-Sexual dysfunction	+	–	+	–	–
-Constipation	+	+	+	+	–
-Diarrhea	+	–	–	–	–
-Hyperhidrosis	–	–	+	–	–
-Orthostatic hypotension	–	–	+	–	–
Cough	+	+	+	+	–
Tremor	+	–	+	+	+
Rigidity	–	–	–	–	–
Ataxia	–	–	–	–	–
Dementia	–	–	–	–	–
Abnormal behavior	–	–	–	–	–
Generalized convulsion	–	–	–	–	–
Disturbance of consciousness	–	–	–	–	–
Encephalitic episode	–	–	–	–	–
Head-MRI					
-Leukoencephalopathy	–	–	–	+	+
-DWI U-fiber high	–	–	–	–	–
Executive function tests					
-MMSE	/	30	30	30	27
-MOCA	/	29	24	30	17
Serum CK (IU/L)	592	102	469	248	182
CSF protein, g/L	/	/	/	/	3.28
CSF cells per mm^3^	/	/	/	/	22,800
Nerve ultrasound	/	Mild thickness in right median and sural nerve	–	–	–

*U, upper limbs; L, lower limbs; D, distal; P, proximal; MRI, magnetic resonance imaging; DWI, diffusion-weighted imaging; MMSE, Mini-Mental State Examination; MoCA, Montreal Cognitive Assessment; CK, creatine kinase; CSF, cerebrospinal fluid; “-”, normal; “↓↓”, absent deep tendon reflex; “↓”, decreased deep tendon reflex; “/”, data not available.*

#### Family 1

Patient 1 (F1-IV:1), who carried 206 repeats, was a 42-year-old male. He initially presented with muscle weakness of four limbs and dysphagia at age 28. Four years later, he developed bladder dysfunction presenting as voiding difficulty, constipation, and diarrhea. He suffered from sexual dysfunction at age 35, and 1 year later, he gradually exhibited numbness in four extremities, paroxysmal irritating dry cough, and postural tremor. By the time he was 41 years old, neurological examination showed his muscle strength indices (Medical Research Council) were grade 4 in the left upper limbs, 4 + in the right upper limbs, 3 + for hip flexion, 5 for knee extension, and 3 for ankle dorsiflexion and plantar flexion. Absent tendon reflex in four limbs and negative Babinski sign were detected. Hypoesthesia of pinprick sensation was noted in the lower limbs. There were, however, no signs of cognitive decline or cerebellar dysfunction.

**FIGURE 3:**
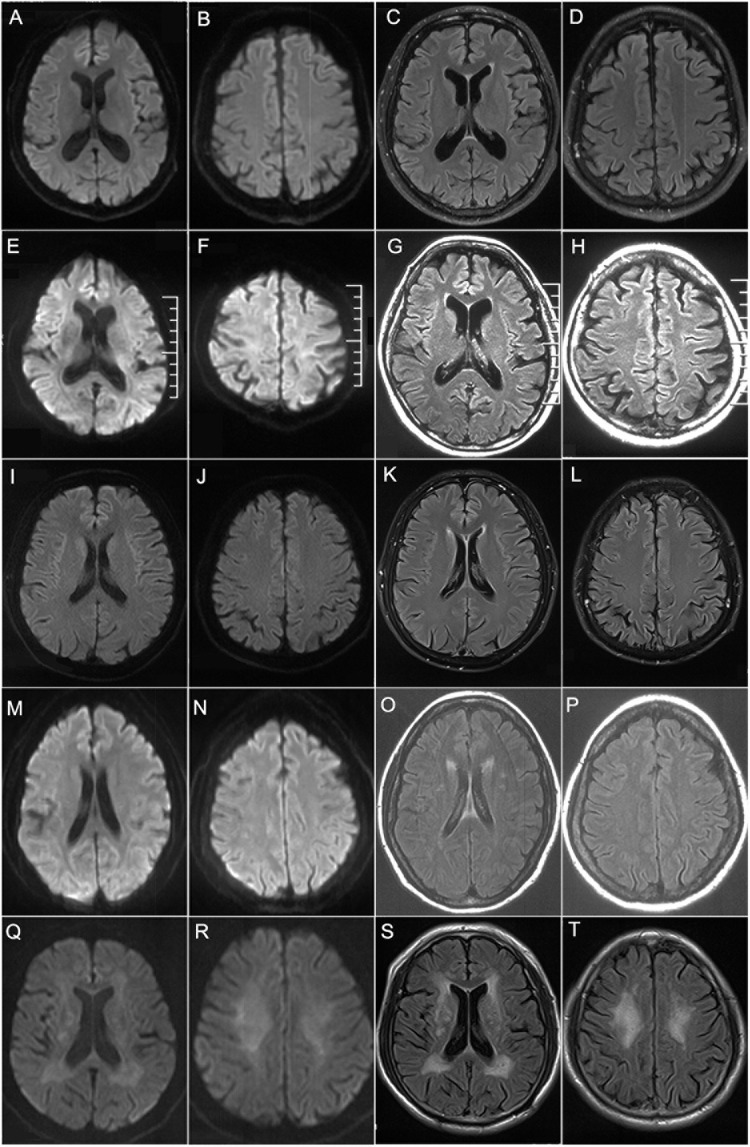
Brain MRI findings. Brain MRIs of patient 1 **(A–D)**, patient 2 **(E–H)**, patient 3 **(I–L)**, patient 4 **(M–P),** and patient 5 **(Q–T)**. **(A–L)** showed no obvious abnormalities on DWI **(A,B,E,F,I,J)** or T2-FLAIR **(C,D,G,H,K,L)**. **(O,P,S,T)** showed high signals along the lateral ventricle on T2-FLAIR while no obvious abnormalities on DWI **(M,N,Q,R)**.

Laboratory examinations revealed that the serum creatine kinase (CK) level was 592 IU/L (normal 25–170 IU/L). Nerve conduction studies (NCS) showed reduction of motor nerve conduction velocity (MCV) and sensory nerve conduction velocity (SCV) in both upper and lower limbs, while compound muscle action potential (CMAP) was decreased predominantly in the lower limbs (Table 2). EMG found neurogenic changes. Brain MRI detected no obvious abnormalities (Figures 3A–D).

**TABLE 2:** Electrophysiological study of patients with abnormal GGC repeat expansions.

**Motor**	**Patient 1**			**Patient 2**			**Patient 3**			**Patient 4**			**Patient 5**		
	**DL (ms)**	**CMAP (mV)**	**MCV (m/s)**	**DL (ms)**	**CMAP (mV)**	**MCV (m/s)**	**DL (ms)**	**CMAP (mV)**	**MCV (m/s)**	**DL (ms)**	**CMAP (mV)**	**MCV (m/s)**	**DL (ms)**	**CMAP (mV)**	**MCV (m/s)**
L Median (<4, >5, >50)	4.50	8.0	44.5	3.23	8.5	45.1	4.29	6.8	50.7	3.23	8.1	47.1	3.73	6.8	47.3
R Median (<4, >5, >50)	3.98	11.6	51.3	4.80	3.5	43.7	4.13	4.5	44.0	3.64	7.2	39.9	3.96	7.1	47.1
L Ulnar (<3, >4, >50)	4.71	2.5	37.0	3.31	6.8	45.9	2.9	7.9	52.5	2.63	4.2	45.1	2.88	8.4	42.8
R Ulnar (<3, >4, >50)	3.56	8.0	43.4	3.68	6.0	48.1	3.19	9.0	41.6	2.67	2.4	39.5	3.20	6.8	47.1
L Peroneal (<5.3, >2, >40)	5.79	0.56	27.6	6.93	0.75	31.4	4.27	1.35	30.1		NR		4.90	2.4	35.9
R Peroneal (<5.3, >2, >40)	4.42	0.64	33.0	5.35	1.94	33.9	5.98	1.37	39.4		NR		4.96	3.4	37.9
L Tibial (<5, >3.5, >40)	4.94	2.0	31.7	5.68	0.65	29.5	4.48	4.8	36.8	6.47	0.56	28.5		ND	
R Tibial (<5, >3.5, >40)	6.18	3.1	33.3	5.60	1.77	32.5	5.98	3.4	36.9	7.49	1.70	22.4		ND	
															

**Sensory**	**Patient 1**		**Patient 2**		**Patient 3**		**Patient 4**		**Patient 5**	
	**SNAP (μV)**	**SCV (m/s)**	**SNAP (μV)**	**SCV (m/s)**	**SNAP (μV)**	**SCV (m/s)**	**SNAP (μV)**	**SCV (m/s)**	**SNAP (μV)**	**SCV (m/s)**

L Median (>5, >50)	NR		7.7	42.4	9.6	49.2	7.3	47.9	6.9	44.1
R Median (>5, >50)	4.5	35.2	3.0	38.1	6.6	52.0	7.4	51.7	8.6	40.5
L Ulnar (>3, >50)	8.5	33.2	7.7	37.2	6.6	40.6	5.3	40.5	NR	
R Ulnar (>3, >50)	3.8	30.8	11.3	39.0	3.5	43.8	5.1	48.2	4.6	38.0
L Radial (>5, >50)	ND		5.7	39.5	ND		ND		ND	
R Radial (>, >50)	ND		3.3	42.1	ND		ND		ND	
L Tibial (>1, >40)	ND		9.0	39.0	NR		NR		7.2	43.9
R Tibial (>1, >40)	ND		5.8	31.7	7.6	44.8	NR		7.1	44.6
L Sural (>1, >40)	6.2	31.9	NR		ND		NR		6.2	42.1
R Sural (>, >40)	6.4	31.7	3.5	32.9	ND		14.5	39.8	NR	

*DL, distal motor latency; CMAP, compound muscle action potential; MCV, motor nerve conduction velocity; SNAP, sensory nerve action potential; SCV, sensory nerve conduction velocity; NR, no response; ND, not detected.*

His mother (F1-III:4) presented with muscle weakness and subsequent muscle atrophy in four limbs at the age of 30 and also developed bladder dysfunction and defecatory dysfunction later on. She died at the age of 53. His grandfather (F1-II:3) died young with no more details. The youngest brother of his grandfather (F1-II:5) developed muscle weakness and atrophy when young and died in his 50 s. The daughter of the elder brother of his grandfather (F1-III:1) showed similar symptoms including muscle weakness and atrophy, but more information on these members was not available. Unfortunately, DNA was not acquired from all the other family members.

#### Family 2

Patient 2 (F2-II:2), who carried 151 GGC repeats, was a 45-year-old female who initially had difficulty walking at 29 years old before she developed irritating dry cough, especially after a long speech. She was then diagnosed with atrophy in bilateral hands and had difficulty extending the toes 5 years later. When she was 39, muscle weakness progressed to the hands and resulted in fine motor impairment such as clamping clothes and making dumplings. She developed bladder dysfunction presenting as voiding difficulty and constipation 3 years later. Upon examination at 41 years old, muscle atrophy in both upper and lower distal extremities was observed. Muscle strength indices were grade 5 in the upper limbs and grade 4 in the lower limbs. Decreased tendon reflex in four limbs and negative Babinski sign were presented. Pes cavus was found in bilateral feet. Hypoesthesia of pinprick sensation was felt in the left foot. Cognitive assessments showed 30 points in MMSE and 29 points in MoCA.

Laboratory examination suggested that the serum CK level was 102 IU/L. Head MRI found no obvious abnormalities (Figures 3E–H). NCS showed reduced MCV and SCV in both upper and lower limbs, while CMAP was decreased predominantly in the lower limbs (Table 2). EMG showed neurogenic changes. Nerve ultrasound revealed mild thickening in the right median and sural nerves.

Her sister (F2-II:4) had similar symptoms of muscle weakness in distal extremities with the onset age of 29 years. She also presented with obvious bladder dysfunction. The size of GGC repeats was 155. However, DNA was not available from the other family members.

#### Family 3

Patient 3 (F3-III:4), who carried 135 GGC repeats, was a 39-year-old male. He complained about numbness in bilateral feet at age 27. Three years later, he suffered from muscle atrophy in bilateral lower limbs and subsequently presented with mild difficulty in walking. At that time, he also developed an irritating dry cough. He was prone to dizziness when standing up and suffered from frequent diarrhea, bladder, and sexual dysfunction. At the age of 37, he was afflicted with paroxysmal muscle weakness in the four limbs, accompanied by palpitation and dyspnea, but the symptoms were relieved 10 min later. Since then, he felt progressive weakness in the distal extremities as well as numbness in the four limbs. He had postural tremor and hyperhidrosis since childhood. Neurological examination at 39 years old showed muscle atrophy in both upper and lower distal extremities. Muscle strength indices were grade 5 in the upper limbs and grade 4 in the lower limbs. Absent tendon reflex in the four limbs and negative Babinski sign were presented. Hypoesthesia of pinprick sensation was also felt in the upper and lower distal limbs. Cognitive assessments showed 30 points in MMSE and 24 points in MoCA.

As for laboratory examination, the serum CK level was 469 IU/L. Head MRI found no obvious abnormalities (Figures 3I–L). NCS showed reduced MCV predominantly in the lower limbs and reduced SCV predominantly in the upper limbs, while CMAP was decreased predominantly in the lower limbs (Table 2). EMG showed neurogenic changes, while nerve ultrasound yielded normal results.

His mother (F3-II:4) had muscle weakness predominantly in the lower limbs as well as constipation in her 30 s and died in her 50 s. Three of the siblings of his mother (F3-II:5, F3-II:7, and F3-II:10) showed similar symptoms of lower limb weakness in their 30 s. One of them (F3-II:7) died in his 50 s. The other two siblings alive also had weakness in the upper limbs. All of the affected individuals in the family had tremor and irritating dry cough, but none of the other family members agreed to the genetic testing.

#### Family 4

Patient 4 (F4-IV:9), who carried 129 GGC repeats, was a 50-year-old female who first complained about muscle weakness of both upper and lower distal extremities at 32 years old. Subsequently, she showed rapid deterioration the next year with proximal muscle weakness in the four limbs. At age 41, she lost the ability to climb stairs or stand from squatting on her own. She developed numbness in the left fingers at 42 years old. By the time she was 47 years old, she began to experience constipation and bladder dysfunction. She developed an irritating dry cough when young and had mild dysphagia and dysarthria. She recalled postural tremor and stomachache since the age of 20. Neurological examination at 42 years old revealed muscle atrophy in the four limbs symmetrically. Muscle strength indices were grade 5 for neck flexors and extensors, grade 5 for shoulder abductors and adductors, grade 5 for elbow flexors, grade 4 for elbow extensors, grade 4 for the distal upper limbs, grade 5 for hip abductors and adductors, grade 4 for hip flexors, grade 4 for knee flexors, grade 5 for knee extensors, and grade 3 for foot dorsal flexors and plantar flexors. Absent tendon reflex in four limbs and negative Babinski sign were presented. Sensation examination showed normal results. She had both resting and postural tremor, mainly in facial and bilateral upper limbs. There were no other signs of cerebellar dysfunction. Cognitive assessments showed 30 points in MMSE and MoCA.

As for laboratory examination, the serum CK level was 248 IU/L. NCS showed decreased MCV in both the upper and lower limbs, and decreased SCV in the lower limbs. Reduced CMAP was observed in the lower limbs (Table 2). Brain MRI revealed leukoencephalopathy along the lateral ventricle without U-fiber high in DWI (Figures 3M–P). Nerve ultrasound showed normal results.

Her sister (F4-IV:7) exhibited similar symptoms of muscle weakness and tremor in her 30s, and gradually developed walking difficulty and severe urinary dysfunction with urinary catheter. The size of GGC repeats was 131. Her father (F4-III:3) showed muscle weakness in the lower extremities, irritating dry cough, and tremor in the hands in his 30s and died at the age of 51. Her grandfather (F4-II:3) had tremor in the hands but no obvious weakness when alive. More detailed information of these members were not available. Neither did we get any DNA from the other family members.

#### The Sporadic Patient

Patient 5 was a 65-year-old male with 126 GGC repeat expansions. Initially, he presented with numbness in the left fingers at 59 years old, which progressed to the whole palm as well as mild muscle weakness. Six months later, muscle weakness and numbness progressed to the right upper limbs and bilateral lower limbs. The symptoms progressively deteriorated, and he had difficulty walking and grasping at the age of 60. He had urinary urgency and incontinence, but without defecatory dysfunction. Additionally, he had a hypertension history of more than 10 years and a diabetes history of 6 years. He suffered from cerebral infarction at age 60 and 64, respectively. After the second cerebral infarction, he presented with obvious memory impairment, and cognitive assessments showed 27 points in MMSE and 17 points in MoCA.

Neurological examination at 60 years old revealed muscle atrophy in bilateral hands, a bit more severe in the left hand. Muscle strength indices were grade 3 in the left proximal upper limbs, grade 4 in the left distal upper limbs, grade 4 in the right proximal upper limbs, grade 5- in the right distal upper limbs, and grade 4 + in the bilateral lower limbs. Hypotonia, absent tendon reflex in the four limbs, and negative Babinski sign were observed. Sensation examination showed hypoesthesia of pinprick sensation in the four extremities, more severe in the left. He had mild postural tremor in bilateral hands.

As for laboratory examination, the serum CK level was 182 IU/L. NCS showed decreased MCV in both upper and lower limbs, and decreased SCV in the upper limbs (Table 2). Brain MRI revealed leukoencephalopathy along the lateral ventricle but without U-fiber high in DWI (Figures 3Q–T). Nerve ultrasound showed normal results. CSF examination showed elevated protein levels (3.28 g/L) and normal leukocyte count (163/mm^3^). CTA revealed multiple cerebral arterial stenosis.

None of his other family members had similar symptoms. RP-PCR did not reveal pathogenic expansion in his son (F5-III:1).

In summary, all the patients showed limb weakness and atrophy in the four extremities, more severe in the distal limbs, as well as absent or decreased deep tendon reflex. Numbness and hypoesthesia of pinprick sensation in the distal extremities were observed in four of these patients, while deep sensation was normal in all the patients. In addition to typical manifestations of HMSN, obvious autonomic impairments including bladder dysfunction and constipation were seen in all the five patients. Notably, four of them (80%) had irritating dry cough, and four (80%) had postural tremor. None of the patients had central nervous system involvement or DWI high-intensity signal along the corticomedullary junction on cerebral MRI (Figure 3). All these patients showed both demyelinating and axonal changes of polyneuropathy, predominantly in the lower limbs and asymmetrically in the upper limbs in the NCS study. Based on the clinical manifestations and electrophysiology studies, all the patients were compatible with the diagnosis of motor–sensory and autonomic neuropathy.

Besides, the clinical manifestations of the other 137 IPN patients without GGC repeat expansions are shown in Supplementary Figure 2. Among them, the male/female ratio was 100:37. The age of onset ranged from 1 to 82 years. The disease duration of 137 IPN patients ranged from 6 months to 40 years.

### Pathological Changes

**FIGURE 4:**
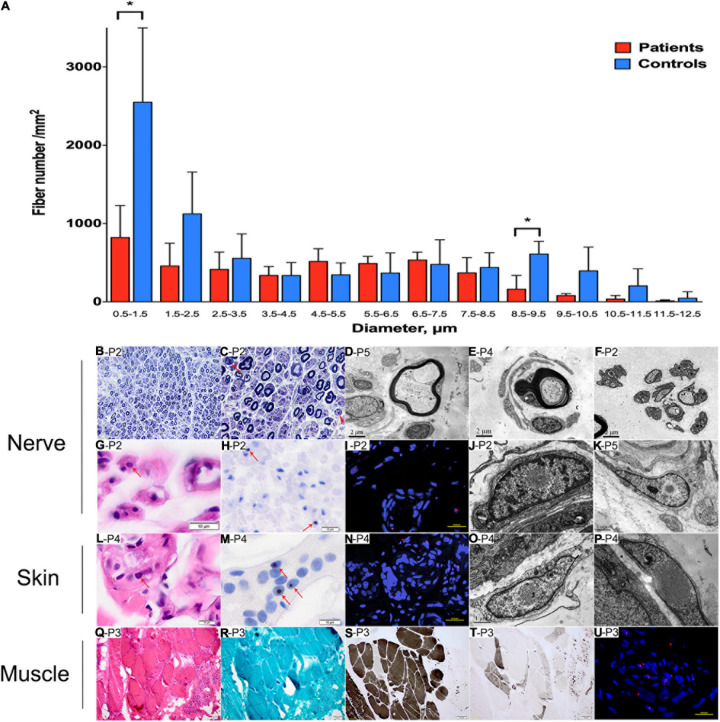
Pathological changes of peripheral nerve, skin, and muscle. **(A)** Distribution pattern of myelinated fibers in patients compared with the control. Data are presented as mean ± SD. Statistical analysis was performed by Student’s t-test. ^∗^*p* < 0.05. (**B–K**, nerve samples; **L–P**, skin samples; **Q–U,** muscle samples) A reduced density of myelinated fibers with thinly myelinated fibers **(B)**, rare axonal degeneration (**C**, arrow) and regeneration clusters (**C**, arrowhead). Thinly myelinated fibers **(D)**, atypical onion-bulb **(E)**, and denervated Schwann cell units **(F)** in EM. Eosinophilic **(G)** and p62-positive **(H,I)** intranuclear inclusions in sural nerve. On EM, intranuclear inclusions containing filamentous aggregates were observed in fibroblast **(J)** and perivascular cells **(K)** in sural nerves. Eosinophilic **(L)** and p62-positive **(M,N)** intranuclear inclusions in skin. On EM, intranuclear inclusions containing filamentous aggregates were observed in fibroblast **(O,P)**. Neurogenic changes **(Q,S,T)** in muscle pathology without rimmed vacuoles **(R)**, and p62-positive **(U)** intranuclear inclusions in muscle. **(B,C)**, toluidine blue staining; **(G,L,Q)**, H&E staining; **(H,M)**, anti-p62 staining; **(S)**, ATP staining (pH 4.4); **(T)**, ATP staining (pH 10.7); **(I,N,U)**, nuclei were counterstained with DAPI. Scale bars: **(B)**, 20 μm; **(C)**, 10 μm; **(D–F)**, 2 μm; **(G,H,L,M)**, 10 μm; **(I,N,U)**, 25 μm; **(J,K,O)**, 1 μm; **(P)**, 0.5 μm; **(Q–T)**, 100 μm. P2, patient 2; P3, patient 3; P4, patient 4; P5, patient 5.

Sural nerve biopsy from patients 2, 3, 4, and 5 revealed that the density of myelinated fibers was moderately or severely decreased to 3,730–5,385/mm^2^ [7,500–10,000/mm^2^ in the control (Jacobs and Love, 1985)] (Table 3). The myelinated fibers with a diameter of 0.5–1.5 and 8.5–9.5 μm were significantly decreased compared with normal controls (*p* = 0.021 and 0.018, respectively) (Figure 4A). Various numbers of thinly myelinated fibers were obviously observed in all the patients (Figure 4B). No patients had obvious onion bulbs. Axonal damages including axonal degeneration and regeneration clusters were occasionally found (Figure 4C). Eosinophilic and p62-positive intranuclear inclusions were detected in all the patients (Figures 4G–I). Besides typical thinly myelinated fibers (Figure 4D), electron microscopy (EM) occasionally revealed some atypical onion bulbs (Figure 4E) and axonal degenerations. Denervated Schwann cell units were also observed (Figure 4F). Intranuclear inclusions containing filamentous aggregates in the fibroblasts or capillary pericytes were found in sural nerves in all the patients (Figures 4J,K).

**TABLE 3:** Pathological findings of patients with abnormal GGC repeat expansions.

	**Patient 2**	**Patient 3**	**Patient 4**	**Patient 5**
Sex	Female	Male	Female	Male
Age at nerve biopsy (years)	41	39	42	60
Density of myelinated fibers (/mm^2^)	3,919	3,871	3,730	5,385
Thinly myelinated fibers	+ +	++	++	+ ++
Onion bulbs	–	–	–	–
Axonal degeneration	+	+	–	–
Regeneration clusters	+	–	+	+
Intranuclear inclusions in EM	+	+	+	+

Skin biopsy from patients 3 and 4 showed eosinophilic and p62-positive intranuclear inclusions in sweat gland cells (Figures 4L–N). EM analysis showed filamentous inclusions in fibroblasts (Figures 4O,P).

Muscle biopsy in patients 3 and 4 showed neuropathic changes including groups of small angular or round atrophy fibers, type grouping, as well as large hypertrophic fibers (Figures 4Q–T). P62-positive intranuclear inclusions were likely to be observed in the atrophic fibers (Figure 4U).

## Discussion

NIID is a heterogeneous disease with highly variable clinical manifestations and has been associated with GGC repeat expansions in the *NOTCH2NLC*. It can be subgrouped according to their dominant phenotype into parkinsonism NIID (NIID-P), dementia NIID (NIID-D), and muscle weakness NIID (NIID-M) (Tian et al., 2019). Patients with NIID-M show muscle weakness that starts from the distal lower limbs and moves up to the throat muscle and face, which is partially similar to the symptoms of IPN patients. Sone et al. (2016, 2019), Ishiura et al. (2019), Tian et al. (2019) have also reported patients with NIID presenting as weakness, sensory disturbance, or autonomic dysfunction previously. Our present study provided more evidence that GGC repeat expansions of *NOTCH2NLC* were associated with IPN.

Based on clinical manifestations, electrophysiological, and pathological studies, it was found that all the five *NOTCH2NLC*-related IPN patients showed a pattern of motor–sensory and autonomic neuropathy. Although the sensory symptoms were mild in some patients, NCS revealed the involvement of both sensory nerves and motor nerves, which is different from dHMN (Liu et al., 2020). More importantly, patients in our cohort had much severe autonomic neuropathy such as bladder dysfunction and constipation, which is very rare in classical CMT (Laurá et al., 2019). While 90.7% of the patients with TTR-FAP showed autonomic dysfunction mainly presenting as gastrointestinal involvement including alternative diarrhea and constipation, orthostatic hypotension and urinary dysfunction, however, were reported in only 27.8% of TTR-FAP patients (Du et al., 2021). Our findings indicated that *NOTCH2NLC*-related IPN was a distinct phenotype of hereditary polyneuropathy. Furthermore, the prolonged distal latency and reduction of MCV and SCV in NCS, as well as thinly myelinated fibers in nerve pathology indicated that the demyelination process was the main pathogenic mechanism of peripheral neuropathy, which is consistent with the findings in previous pathological studies of NIID patients (Sone et al., 2005). The reduction of CMAP in most patients suggested that axonal degeneration was partly involved in the pathogenesis of peripheral neuropathy. In addition, the muscle pathology that showed some similarities to that of spinal muscle atrophy (SMA) (Buchthal and Olsen, 1970), as well as the spontaneous activity in EMG with normal CMAP in NCS in patient 5 suggested the possibility of involvement of anterior horn cells in the spinal cord. Recent studies showed that RNA toxicity and toxic polyglycine (polyG) protein of pathogenic GGC repeat expansion could be involved in the pathogenesis of NIID (Boivin et al., 2021; Deng et al., 2021; Yu et al., 2021). Nevertheless, further studies are needed to elucidate the pathological mechanisms by which peripheral nerves are damaged.

The finding related to intranuclear inclusions in nerve, muscle, and skin tissues indicated that *NOTCH2NLC*-related IPN was a multisystem degenerative disease. Intriguingly, most *NOTCH2NLC*-related IPN patients had chronic dry cough in addition to typical symptoms of peripheral neuropathy. The underlying mechanism of dry cough might be interpreted as follows. (1) Irritating dry cough was reported to be as an initial symptom that accounted for 51% of NIID patients in a cohort study (Chen et al., 2020). Based on the findings that intranuclear inclusions were found in multiple organs including the lungs (Sone et al., 2005), it could be assumed that intranuclear inclusions could possibly exist in the trachea and bronchia, which might be related to the dysfunction of the airway. (2) Chronic dry cough had been reported in hereditary sensory neuropathy (Spring et al., 2005; Barros et al., 2014) and familial amyloid polyneuropathy (Yuan et al., 2019). Considering the severe autonomic symptoms and denervated Schwann cell units on EM in these patients, the cough might have partly arisen from the damages to C or Aδ unmyelinated fibers in peripheral nerves that constructed the autonomic nerve networks of the airways for cough reflex hypersensitivity (Canning, 2006; Gibson and Vertigan, 2015). It could be concluded that cough was not necessarily specific to *NOTCH2NLC*-related IPN patients. More patients are needed to elucidate the sensitivity and specificity of dry cough for *NOTCH2NLC*-related IPN in future studies.

Postural tremor was another frequent concomitant symptom in this *NOTCH2NLC*-related IPN patient group. It was still open for discussion whether the tremor originated from the cerebellar or extrapyramidal involvement (Sun et al., 2020), or was associated with peripheral neuropathy itself (Hong et al., 2016). Additionally, there were two patients who showed white matter lesions along the lateral ventricle in brain MRI. However, none of these patients had other signs related to the central nervous system, including dementia, encephalitic episode, and typical MRI features of adult-onset NIID (Lu and Hong, 2021). Whether *NOTCH2NLC*-related IPN and NIID could be included in a broad phenotypic spectrum requires long follow-up study to observe the overall symptoms along the disease progression. However, these extra-neuropathy symptoms might be some of the important indicators to diagnose *NOTCH2NLC*-related IPN as early as possible.

Notably, the GGC repeat expansion in *NOTCH2NLC* was associated with oculopharyngodistal myopathy (OPDM) recently (Ogasawara et al., 2020; Yu et al., 2021). OPDM is a rare adult-onset hereditary muscle disease clinically characterized by progressive ocular, pharyngeal, and distal limb muscle involvement and pathologically by rimmed vacuoles in muscle fibers. Patients in our group showed limb weakness without ocular and facial involvement, and no myopathic changes were found in EMG and muscle pathology, which suggested that *NOTCH2NLC*-related IPN was different from *NOTCH2NLC*-related OPDM.

Studies, so far, have showed an ambiguous tendency between the length of GGC repeat expansion in *NOTCH2NLC* and the variable phenotypes (Westenberger and Klein, 2020). NIID-M patients were found to harbor longer GGC repeats than those with essential tremor, NIID-P, or NIID-D (Tian et al., 2019; Westenberger and Klein, 2020). However, the repeat number of GGC in *NOTCH2NLC*-related IPN is smaller than that in NIID-M (126-206 vs. 118-517), but patients have relatively early age of onset, suggesting that there is weak correlation between the size of the GGC repeats and the clinical symptoms. Moreover, in the present study, some differences in clinical manifestations were observed in these five *NOTCH2NLC*-related IPN patients, including onset age, severity and distribution of muscle weakness, sensory symptoms, and autonomic symptoms. However, we could not conclude the relationship between the GGC repeat size and phenotype variation considering the very small sample size of this patient group. Additionally, the genetic background, and modifying and environmental factors may also contribute to the phenotypic heterogeneity.

In summary, the GGC repeat expansion in the *NOTCH2NLC* is associated with a part of inherited peripheral neuropathy, which expands the phenotype of the *NOTCH2NLC*-related repeat expansion spectrum. Significantly, screening of GGC repeat expansions in the *NOTCH2NLC* should be considered in motor–sensory and autonomic neuropathy with yet undetermined genetic causes, especially concomitant with tremor or irritating dry coughing.

## Data Availability Statement

The data supporting the results of this study are available from the corresponding author upon reasonable request.

## Ethics Statement

Written informed consent was obtained from the individual(s) for the publication of any potentially identifiable images or data included in this article.

## Author Contributions

HW, LM, ZW, and YY designed the studies. HW, JY, and JD completed the genetic analysis and analysis of the data. JY contributed to the PCR methods. WZ, HL, and YY evaluated the muscle pathology. MY, XS, WL, and ZJ carried out and evaluated the electrophysiological examination. WZ, HL, LM, and ZW contributed to the clinical diagnosis of the patients. LM and JL contributed to the nerve, muscle, and skin biopsy of the patients. HW, DH, ZW, and YY wrote and edited the manuscript. YY supervised the project. All authors read and approved the final manuscript.

## Conflict of Interest

The authors declare that the research was conducted in the absence of any commercial or financial relationships that could be construed as a potential conflict of interest.

## Supplementary Material

Supplementary Figure 1 The distribution of repeat length in 103 IPN patients without abnormal GGC repeats in *NOTCH2NLC* and 100 normal controls.

Supplementary Figure 2 Clinical manifestations of 137 IPN patients without abnormal GGC repeats in *NOTCH2NLC*.

## Abbreviations

AL-PCR: fluorescence amplicon length analysis-PCR
ATPase: adenosine triphosphatase
CMAP: compound muscle action potential
CMT: Charcot-Marie-Tooth disease
dHMN: distal hereditary motor neuropathy
DWI: diffusion-weighted imaging
EM: electron microscopy
HMSN: hereditary motor and sensory neuropathy
HSAN: hereditary sensory and autonomic neuropathy
IPN: inherited peripheral neuropathy
MCV: motor nerve conduction velocity
mGT: modified Gomori trichrome
MMSE: the Mini-Mental State Examination
MoCA: the Montreal Cognitive Assessment
MRI: brain magnetic resonance imaging
NADH-TR: NADH-tetrazolium reductase
NCS: nerve conduction studies
NGS: next-generation sequencing
NIID: neuronal intranuclear inclusion disease
RP-PCR: repeat-primed polymerase chain reaction
SCV: sensory nerve conduction velocity
SFN: small fiber neuropathies
SMA: spinal muscle atrophy
SNAP: sensory nerve action potential
TTR-FAP: transthyretin familial amyloid polyneuropathy
WES: whole-exome sequencing
WGS: whole-genome sequencing.
