# Supplementary figures and images for: GGC Repeat Expansion in the NOTCH2NLC Gene Is Associated With a Phenotype of Predominant Motor–Sensory and Autonomic Neuropathy
Source: Front Genet. 2021 Jul 7;12:694790. doi: 10.3389/fgene.2021.694790 (PMC8293674; doi:10.3389/fgene.2021.694790)

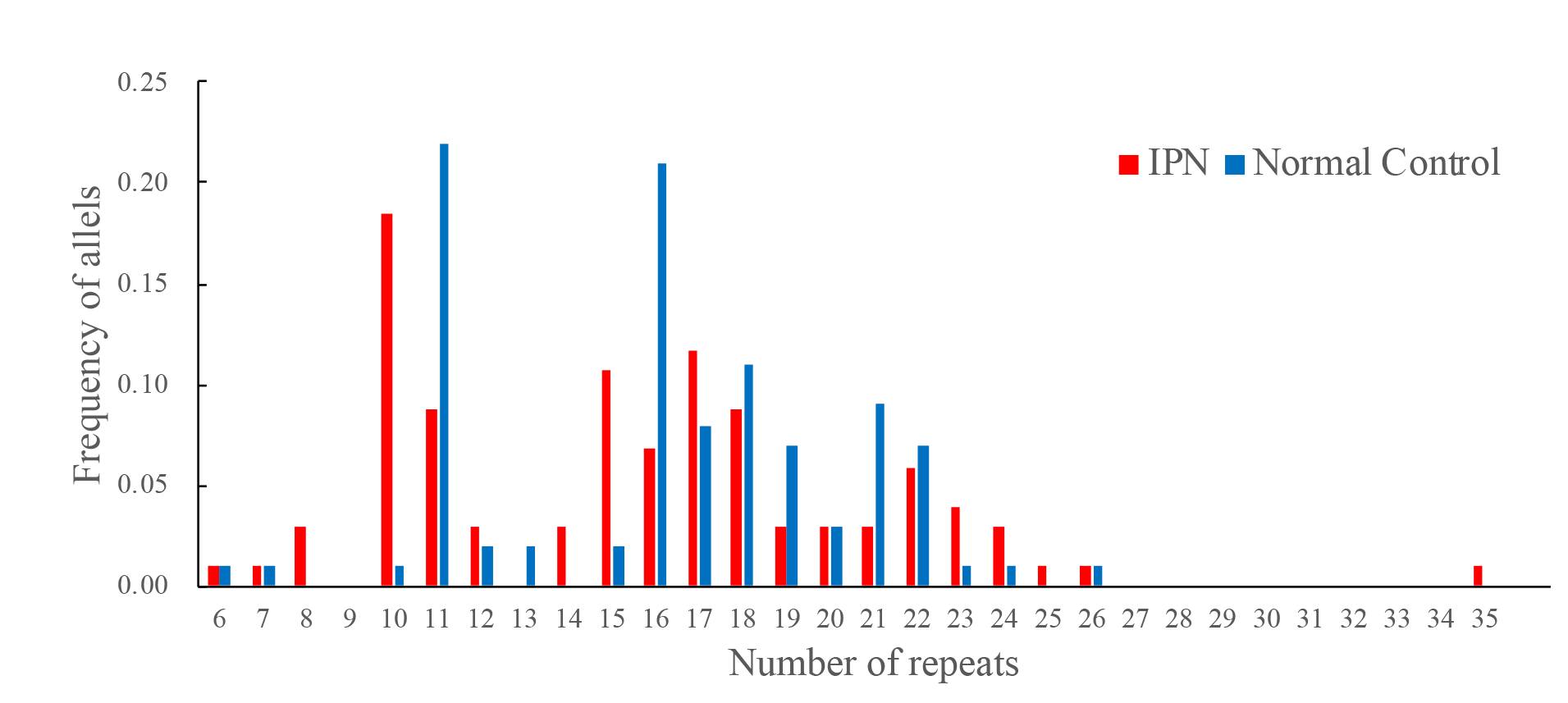

Supplement: Supplementary Figure 1 — The distribution of repeat length in 103 IPN patients without abnormal GGC repeats in NOTCH2NLC and 100 normal controls. [file Image_1.TIF]

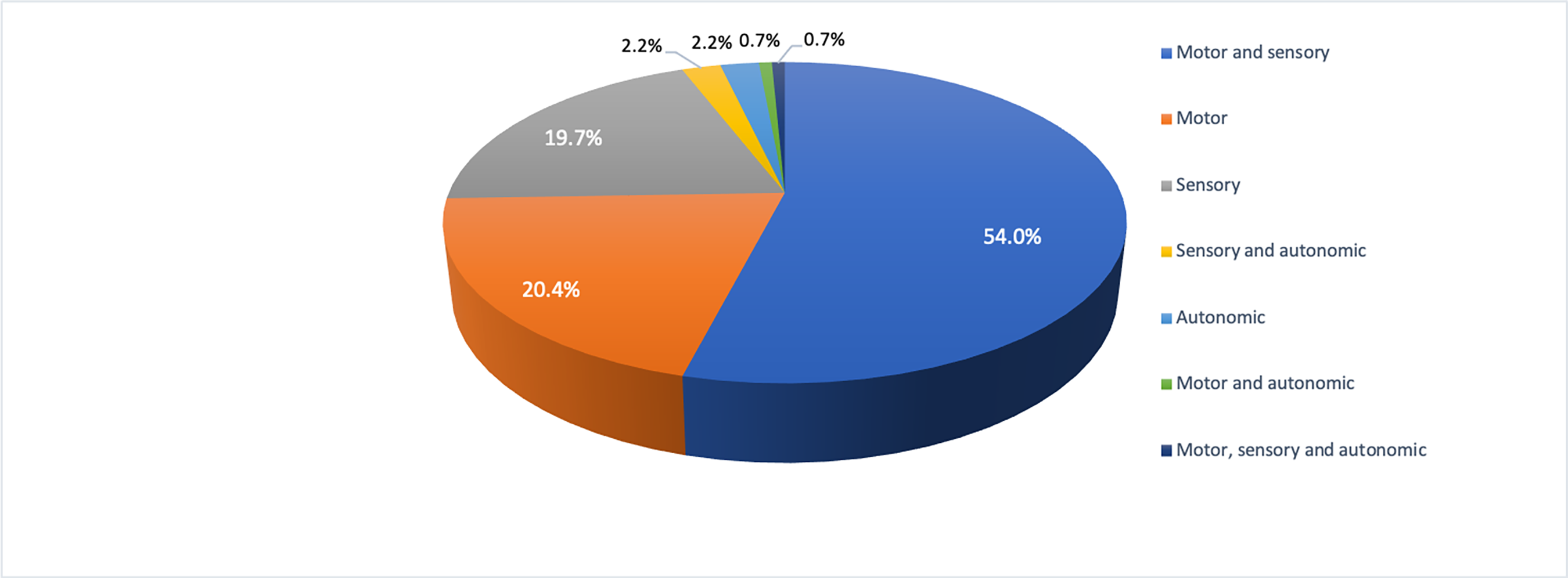

Supplement: Supplementary Figure 2 — Clinical manifestations of 137 IPN patients without abnormal GGC repeats in NOTCH2NLC. [file Image_2.TIF]
